# The bZIP transcription factors in *Liriodendron chinense*: Genome-wide recognition, characteristics and cold stress response

**DOI:** 10.3389/fpls.2022.1035627

**Published:** 2022-11-07

**Authors:** Mingyue Li, Delight Hwarari, Yang Li, Baseer Ahmad, Tian Min, Wenting Zhang, Jinyan Wang, Liming Yang

**Affiliations:** ^1^ College of Biology and the Environment, Nanjing Forestry University, Nanjing, China; ^2^ Innovation Center of Excellence, Jiangsu Academy of Agricultural Sciences, Nanjing, China; ^3^ School of Food and Biological Engineering, Jiangsu University, Zhenjiang, China

**Keywords:** *Liriodendron chinense*, bZIP transcription factor, phylogenetic analysis, genomic survey, cold stress response

## Abstract

The basic leucine zipper (bZIP) is a transcription factor family that plays critical roles in abiotic and biotic stress responses as well as plant development and growth. A comprehensive genome-wide study in *Liriodendron chinense* was conducted to identify 45 bZIP transcription factors (LchibZIPs), which were divided into 13 subgroups according the phylogenetic analysis. Proteins in the same subgroup shared similar gene structures and conserved domains, and a total of 20 conserved motifs were revealed in LchibZIP proteins. Gene localization analysis revealed that LchibZIP genes were unequally distributed across 16 chromosomes, and that 4 pairs of tandem and 9 segmental gene duplications existed. Concluding that segmental duplication events may be strongly associated with the amplification of the *L. chinense* bZIP gene family. We also assessed the collinearity of LchibZIPs between the *Arabidopsis* and *Oryza* and showed that the LchibZIP is evolutionarily closer to *O. sativa* as compared to the *A. thaliana.* The *cis-*regulatory element analysis showed that LchibZIPs clustered in one subfamily are involved in several functions. In addition, we gathered novel research suggestions for further exploration of the new roles of LchibZIPs from protein-protein interactions and gene ontology annotations of the LchibZIP proteins. Using the RNA-seq data and qRT-PCR we analyzed the gene expression patterns of LchibZIP genes, and showed that LchibZIP genes regulate cold stress, especially LchibZIP4 and LchibZIP7; and LchibZIP2 and LchibZIP28 which were up-regulated and down-regulated by cold stress, respectively. Studies of genetic engineering and gene function in *L. chinense* can benefit greatly from the thorough investigation and characterization of the *L. chinense bZIP* gene family.

## Introduction

The basic leucine zipper (bZIP) family is one of the most extensive and varied families of transcription factor (TF) genes in plants (Liu et al., 2014). Regulating numerous biological processes, including plant development, senescence, light signaling, pathogen defense, and reactions to diverse environmental stimuli, depend on (Van Leene et al., 2016; Liu et al., 2020a). bZIP TFs are characterized by a conserved bZIP domain, spanning 40–80 amino acids (Castro et al., 2017). The conserved domain comprise of two major components, a base amino acid region binding to a specific DNA sequence, an amphipathic alpha helix consisting of one or more heptapeptide repeats, and a leucine zipper domain involved in oligomerization (Gai et al., 2020). The bZIP TFs primarily bind to *cis*-elements with the ACGT core motif, such as the A-box (TACGTA), C-box (GAC GTC), G-box (CACGTG), and ABRE (CC-ACGTGG) (Manzoor et al., 2021).

Low temperature stress as one of the main limiting factors of plant growth, affect the geographic distribution of plants, plant total yield, and growth, and development (Wang et al., 2018). Nonetheless, plants have developed special bio-chemical mechanisms to improve low temperature resistance (Hwarari et al., 2020b). Various cold-responsive genes are expressed in reprogrammed transcriptome patterns to modify the characteristics of cell walls and plasma membranes as well as countless physiological and biochemical processes such as calcium fluxes and membrane integrity (Venkatesh et al., 2020; Wang et al., 2020). Besides interacting with the promoter or enhancer region of DNA, plant TFs induce and activate transcription and expression of regulatory genes. Previous studies have shown that bZIP TFs play a significant role during low temperature stress regulation. O wheat bZIP TaABL (ABI-like), and increased cold resistance in wheat (Cai et al., 2018). In Arabidopsis, CsbZIP18 has also been shown to negatively regulate cold tolerance through the ABA-dependent pathway (Yao et al., 2020). In *Brachypodium*, 75% of the BdbZIP genes were exhibited to interact with ABA-responsive *cis*-elements (ABRE) and regulate drought responsive genes, consecutively enhancing drought tolerance (Liu and Chu, 2015). GmbZIP19 has also been labelled as a potent inhibitor of salt and drought tolerance and a positive regulator of disease resistance (He et al., 2020). These findings evidence the roles of the bZIP gene family involvement during abiotic stress regulation.

Functional characterization of the bZIP transcription factors has been done in various plant species including: rose (Li et al., 2022b), and watermelon (Unel et al., 2019). Although, little is known about their origins, properties, and functions in L. *chinense*. *L. chinense* belongs to the Magnolia L clade, and is one of the most significant lumber species in the world; known for its great commercial value and dispersion (Yin et al., 2021). However, it is affected by abiotic stresses including low temperature stress, which adversely reduce quality and yield (Chen et al., 2019; Cao et al., 2022). Here, we described the bZIP genes family in *L. chinense* based on their chromosomal position, gene duplication, *cis*-regulatory elements, gene structure, conserved motifs, evolutionary relationships, expression profiles, protein-protein interactions, and gene ontology annotation. In addition, we investigated the effects of low-temperature stress on the LchibZIP genes, and confirmed their expression patterns using the transcriptome and qRT-PCR analyses. The results will provide a systematic explanation for the evolution and conservation of the LchibZIP genes and lay a foundation for further functional research.

## Materials and methods

### Documentation of bZIPs in *L. chinense*

*L. chinense* protein sequences were retrieved from the local protein database and *A. thaliana* bZIP protein sequences were mined form the TAIR (https://www.Arabidopsis.org/). To find probable *L. chinense* bZIP family members, potential proteins were identified using the local BLASTP algorithm and HMMER software (E-value<1e^−5^). Candidate bZIP protein sequences were screened according to their conserved domains in the databases: NCBI Conserved Domain Search (CDD) (https://www.ncbi.nlm.nih.gov/Structure/cdd/wrpsb.cgi) and Protein family (Pfam) (http://pfam.xfam.org), and redundant, incomplete sequences were removed. To explore the evolutionary relationship of the bZIP gene family among land plants, authenticated bZIP protein sequences of 8 land plants: *C. reinhardtii, P. patens, S. moellendorfii, P. abies, M. acuminata, O. sativa, P. trichocarpa*, and *V. vinifera* were mined from the online plant transcription factor database (http://planttfdb.gao-lab.org/). The ExPASy online tool (https://www.expasy.org/vg/index/Protein) was used to determine the physicochemical properties of identified LchibZIP members.

### Multiple sequence alignments and phylogenetic analysis

Multiple sequence alignments were performed on the full bZIP protein sequences retrieved above (in *L. chinense, C. reinhardtii, P. patens, S. moellendorfii, P. abies, M. acuminata, O. sativa, P. trichocarpa*, and *V. vinifera*) with MUSCLE program and default parameters as implemented in MEGA 11 software. Subsequently, MEGA 11 software was used to construct a phylogenetic tree based on the alignments using the neighbor-joining tree (NJT) method, replicated 100 times using the p-distance model. To confirm the result from the NJT method, another phylogenetic tree was constructed using the Maximum likelihood (ML) method.

### Exon and intron structures, and conserved motifs in LchibZIPs

Using MEME, conserved motif analysis of the LchibZIP protein sequences was carried out to support the phylogenetic analyses. The motif width ranged from 10 to 100 amino acids, and the number of pixels was 20. The Gene Structure Display Server (http://gsds.cbi.pku.edu.cn/) was used to analyze the gene structure of the bZIP gene family (Li et al., 2011).

### Chromosome distribution, collinearity analysis, and Ka/Ks of bZIPs in *L. chinense*

The genome sequence of *L. chinense* was used to establish the location of the LchibZIP gene on each chromosome, then the Tbtools software was used to show the localization of each LchibZIP gene member. *L. chinense, Oryza*, and *Arabidopsis* homologous gene pairs were searched using the BLASTP software, and the Ka, Ks, and Ka/Ks values were computed using the TBtools built-in Ka/Ks Calculator based on their gene pairs. Gene collinearity with *Arabidopsis* and *Oryza* was built and illustrated in relation to *L. chinense* (Hu et al., 2022).

### Prediction of *cis*-regulatory elements

To predict the *cis*-acting elements present in each LchibZIP gene, the Tbtools was utilized to extract the promoter region 2 kb upstream of the CDS; consequently, the sequences were then uploaded to the PlantCare website. The outcome was sorted and condensed for analysis, and viewed using the Tbtools.

### Gene ontology (GO) analysis of the LchibZIPs

To gain an insight of the potential biological roles of the identified LchibZIP genes, the Blast2GO v5.2.5 was used to predict and categorize the LchibZIPs according to function. All the investigated gene were assigned to three categories of GO terms: biological process (BP), molecular function (MF), and cellular component (CC) depending on potential function.

### Prediction of LchibZIP protein interaction network

Protein interaction analysis provides a base for predicting protein functionality. To full comprehend the possible protein interconnection between the LchibZIP members, the String *v*10 online database (https://string-db.org/) was used to construct a protein network based on *A. thaliana* homologs. *A. thalina* homologs were retrieved through the online BLASTP search (https://blast.ncbi.nlm.nih.gov/Blast.cgi).

### Plant material treatment and qRT-PCR analysis

The *L. chinense* seedlings were incubated in a greenhouse with natural light, 70% humidity, and a 25°C temperature, while those at 3 months were kept at 4°C. Leaves of seedlings were sampled at 0, 1, 3, 6, 12, 24 h and 3 d after the experiment treatment, and three biological replicates were performed. The samples mentioned above were subjected to transcriptome sequencing. The results of the self-sequencing were used to derive the transcriptome information for LchibZIPs. The expression profiles of each LchibZIP member at each stage under low-temperature treatment were displayed as a heatmap. Afterwards, total RNA was extracted from *L. chinense* seedlings treated at 0, 6, 24 h and 3d using a chloroform-free plant total RNA extraction kit from BioTeke Corporation and subjected to qRT-PCR analysis. Primer5.0 was used to create all of the primers for qRT-PCR (Li et al., 2022a; Hwarari et al., 2022a).

## Results

### Identification of bZIPs in *L. chinense*

To identify bZIP genes in *L. chinense*, the Pfam (PF00170) database was used to search and authenticate bZIP protein sequences in the *L. chinense* protein database. To verify the bZIP conserved domain in potential members, the CDD database in NCBI was searched. After removing repeated sequences, 45 *LcbZIP* genes were identified, and annotated as LcbZIP1 through to LcbZIP45 (**Supplementary Table S1**). The length of the identified LchibZIP members ranged from 121 (LchibZIP25) to 881 (LchibZIP36) amino acids, with an average protein length of 338 amino acids. The molecular weight ranged from 13404.88 (LchibZIP35) to 64715.15 (LchibZIP45) Da, and the isoelectric points (pI) ranged between 4.7 (LchibZIP10) and 9.83 (LchibZIP25), suggesting that LchibZIPs are weakly basic.

**Figure 1:**
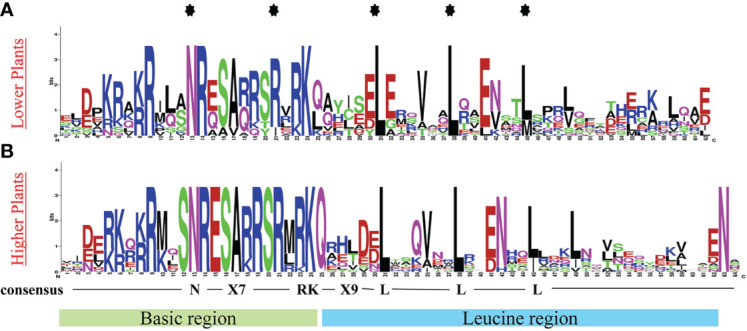
Conserved bZIP domain logo in **(A)** Lower plants, using protein sequence representatives from: *C. reinhardtii* and *P. patens*, **(B)** Higher plants, using protein sequence representatives from 5 land plants. The logo depicts the basic region (motif 2-20) and the leucine zipper region (motif 20-35). The logo was generated using online WebLogo tool (https://weblogo.berkeley.edu/logo.cgi).

To fully understand the structure of identified bZIP protein conserved domain, we investigated for the conserved domain using the online webLogo tool (https://weblogo.berkeley.edu/logo.cgi). Presentative sequences from each plant showed a basic region in the N-terminal, composed of N-(X)7-RK; a Leucine rich motif (zipper domain) in the C-terminal with a notable heptapeptide repat of Leucine (L) and related hydrophobic amino acids (**Figure 1**).

### Phylogenetic analysis of the LchibZIPs

**Figure 2:**
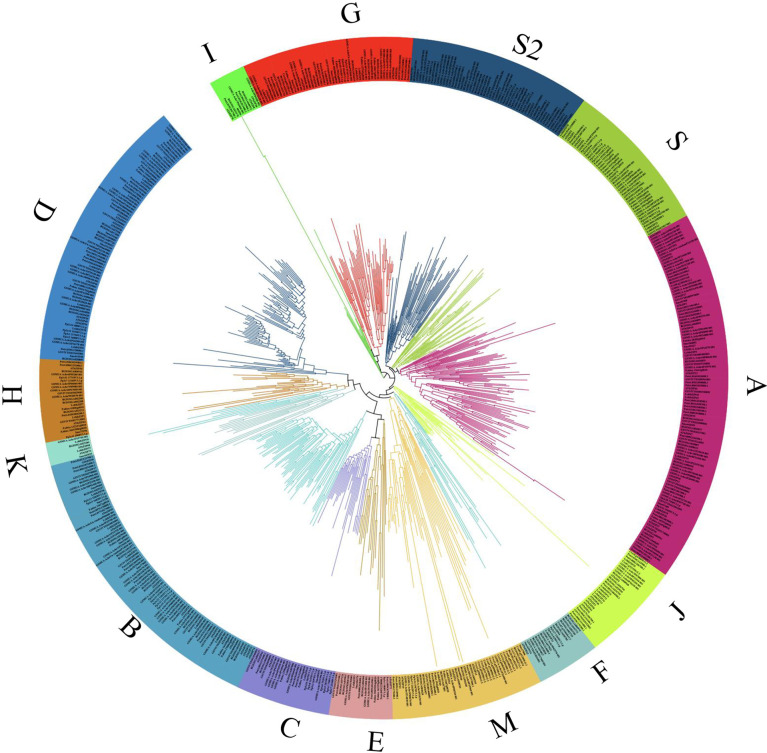
The bZIP gene family, phylogenetic tree analysis of 10 different plant species. The tree was constructed from 618 full bZIP protein sequences using MEGA X software, with 1000 bootstrap value and default parameters. Different color schemes shown represent cluster groups denoted by alphabetic letters (A-K, M, and S).

**Figure 3:**
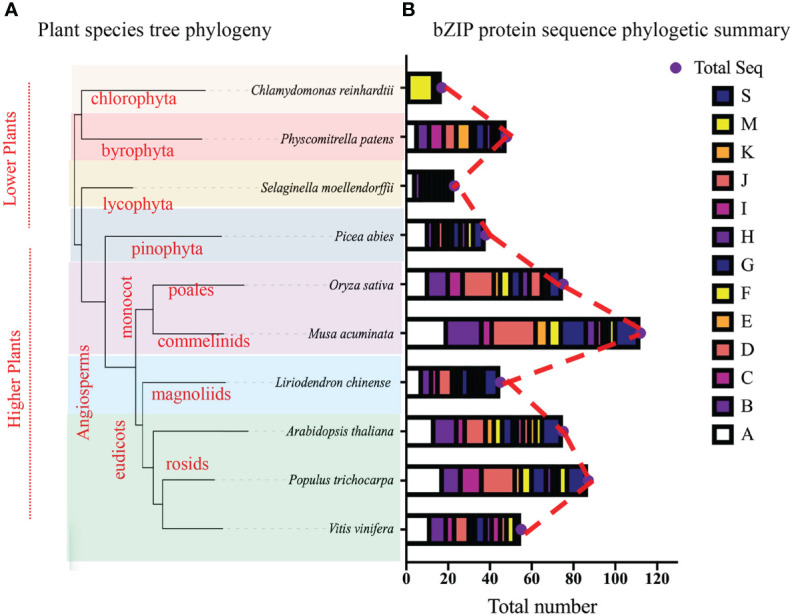
The bZIP phylogenetic tree, **(A)** Interspecies phylogetic tree, beautified using the online iTOL tree software, phylogenetic tree was constructed using the Xshell ortho-finder software. Varying color schemes show different plant clades. **(B)** Summary of the bZIP phylogenetic tree from **Figure 2**, total number of proteins present in each group ae shown using the bar charts. Different color schemes shown represent cluster groups denoted by alphabetic letters (A-K, M, and S) as depicted by the key.

To explore the evolutionary relationship among bZIP members, we constructed a Maximum Likelihood (ML) phylogenetic tree using Mega X software (**Figure 2**; **Supplementary Figure S1**). A total of 618 full bZIP protein sequences from 10 plant species were divided into 14 clusters (A-K, M, S and S2), based on protein structure, arrangement and possibly protein function. Several protein sequences among the analyzed lot, were evolutionary independent and were assigned to cluster M and S, concurring to previous publications (Izawa et al., 1993; Jakoby et al., 2002). Almost, all the plant species were fully represented in individual clusters, except for *C. reinhardtii* which clustered in cluster M. Specifically, the LchibZIP gene family was fully represented in all groups except group I suggesting that the *L. chinese* bZIP gene family is evolutionary conserved. Deeper analysis showed that cluster D, H, K, B, C and E belonged to the same paraphyly group, suggesting that the bZIP sequences diverged for the same common ancestors. In addition, group A and I had the most (67) and the least (10) number of bZIP sequences (**Figure 3B**).

To fully comprehend the evolution of the bZIP gene family, we constructed an interspecies phylogenetic tree using Xshell software (**Figure 3A**). Findings exhibited that all these plant species diverged from a common ancestor, and speciation events led to species diversification. Specifically, the chlorophyte (*C. reinhardtii*), the bryophyte (*P. patens*), and the lycophyte (*S. moellendorfii*) were shown to have diverged earlier suggesting that, they predate evolution and that bZIP gene family can be traced back early life. In comparison, the higher plants had the most recent divergence, this may be related to the angiosperm evolution and expansion. In total, our findings suggest that the *L. chinense* bZIP gene family is evolutionary conserved and can be traced back the ancient common ancestor in the earlier periods.

### Gene structure and motif analyses of LchibZIPs

**Figure 4:**
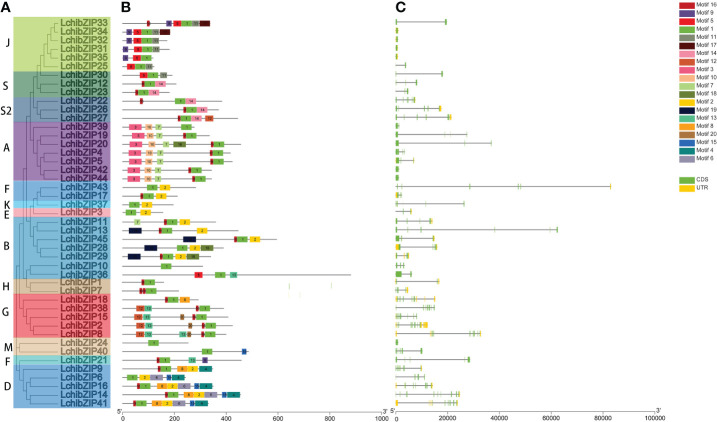
**(A)** phylogenetic relationship **(B)** gene structure and **(C)** conserved motifs of LchibZIPs. The gene structure dynamics of LchibZIPs were predicted using GSDS software. Exons are indicated by green boxes and introns are indicated by black lines. Conserved motifs were scanned with MEME. Different motifs are represented by different colored boxes.

The bZIP domain is essential for binding to the DNA of other TFs, and presence of multiple conserved motifs outside of the bZIP domain may suggest increased functionality. To understand conserved motif arrangement and distribution in each LchibZIP protein, we searched the online MEME tool. A total of 20 conserved motifs were identified at an E-value of less than 10 (**Figure 4B**; **Supplementary Figure S2**). Results showed that individual group clusters of bZIP proteins had similar motif arrangement and distribution, and clustered in consistent with the LcbZIP phylogenetic analysis (**Figure 4A**). In addition, motif 1 was present in all the LchibZIPs, which we concluded to be the bZIP domain. Groups B, D, E, F, and K, all shared motif 2. The majority of the conserved motifs were present in different groups. For instance, motif 3, motif 10, and motif 7 was only found in group A, motif 6 and 4 were only found in group D, and motif 19 was only found in group B. Implying that the LcbZIPs within these groups have particular and distinct roles.

To have a full comprehension of the LchibZIP gene structure, we examined the intron-exon arrangement of 45 LcbZIPs. Five (11.1%) of the 45 LchibZIP genes lacked the introns, and this condition was noticed in groups J and B only. The LchibZIP gene exon count ranged from 1 to 10, demonstrating the great level of differentiation in the 45 LchibZIP genes. However, within a single subgroup, the exon-intron structure of genes was more conserved. For example, most genes with consecutive open reading frames (ORFs) were found in group A, and group A carried one to four exons. Gene structures among members of the same group are frequently similar (**Figure 4C**).

### Chromosome distribution of bZIPs in *L. chinense* genome

**Figure 5:**
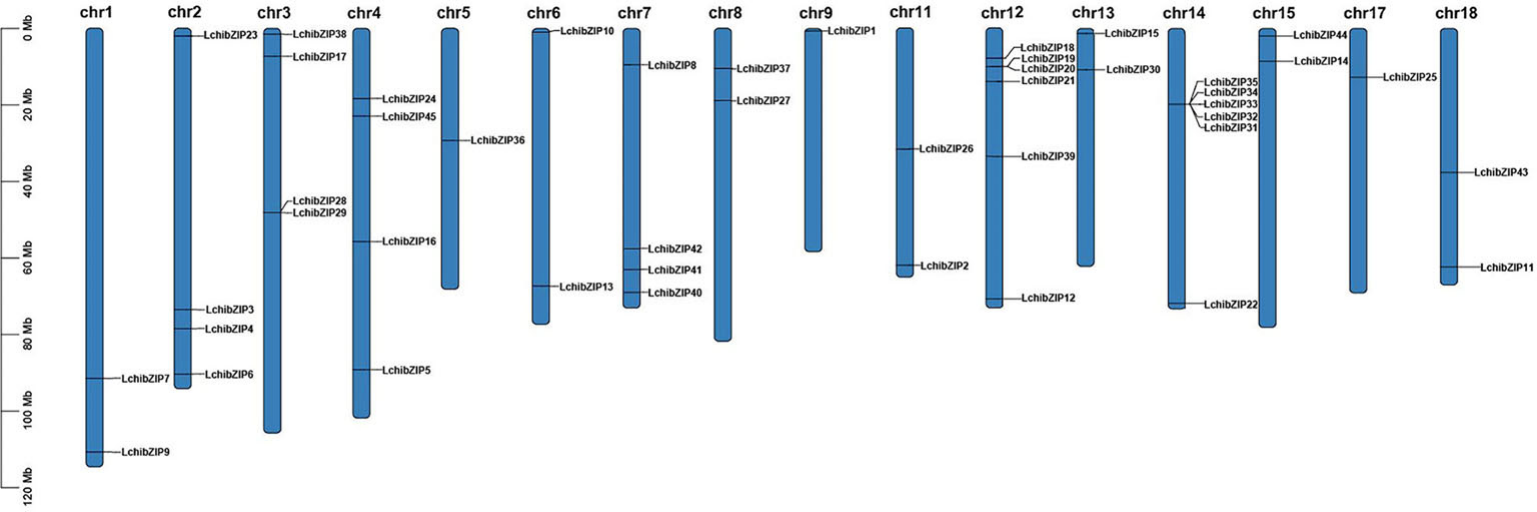
Distribution of 45 bzip genes on 16 chromosomes of *L. chinense*. Chromosome lengths and gene positions can be inferred from the scale on the left.

To determine gene location and gene duplication of LchibZIPs, we searched the chromosomal distribution of the bZIP gene family using data from the *L. chinense* genome annotation and TBtools (**Figure 5**). 45 bZIP genes were found to be unevenly distributed throughout 16 of the 19 chromosomes in the *L. chinense* genome, and were absent on Chr10, Chr16 and Chr19. Five genes were localized on Chr1, Chr4, Chr6, Chr7, and Chr11, and two on Chr12 and Chr14

### Collinear correlations of bZIPs in *L. chinense*, *Arabidopsis*, and *Oryza*

Analysis for gene collinearity and duplication provides a basis for understanding gene expansion and evolution, and one of the main forces behind the genome and genetic systems evolution is gene replication. Plant gene families have expanded mostly as a result of tandem and segment repetitions. We performed a collinearity analysis in the *L. chinense* genome, and showed that segmental duplications occurred on 10 distinct genetic pairs across 14 of the 19 chromosomes (**Supplementary Figure S3**). Additionally, we found only four pairs of tandem duplication genes (LchibZIP19/LchibZIP20, LchibZIP28/LchibZIP29, LchibZIP31/LchibZIP32, and LchibZIP34/LchibZIP35) in the *L. chinense bZIP* genome. Interestingly, tandem duplication events of *LchibZIPs* were relatively infrequent compared to segmental duplication events (**Figure S3**). These findings suggest that gene duplication plays a significant role in the growth of the *bZIP* gene in *L. chinense* and that segment duplication events are the primary drivers behind *LchibZIP* gene evolution, and contributed to the expansion of the LchibZIP gene family.

**Figure 6:**
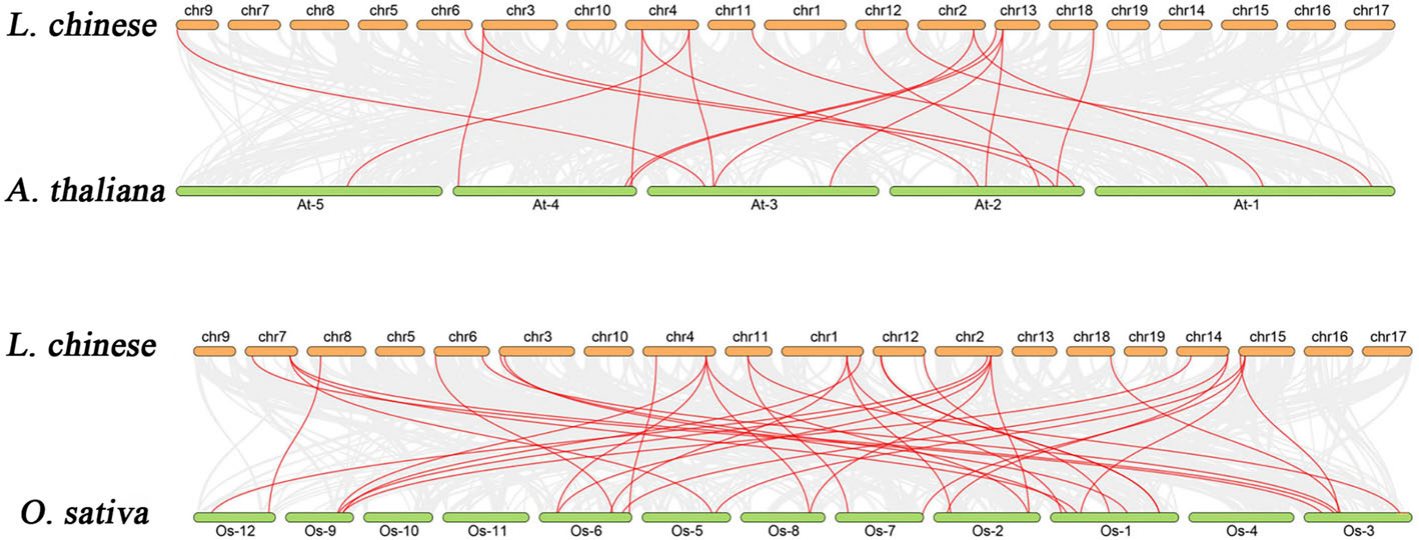
Analysis of the collinear relationship between the bzip gene of *L. chinense* and the bzip gene of *Arabidopsis* and *Oryza*.

To identify orthologs between the *L. chinense* bZIP genes and two other plant species: (*Arabidopsis thaliana*) and monocot (*Oryza sativa*), we performed the collinearity correlation analysis (**Figure 6**). There were 18 orthologous gene pairs between *L. chinense* and *Arabidopsis*, and 35 between *L. chinense* and *Oryza*. We concluded that *L. chinense* and *Oryz*a share more common gene pairs than *L. chinense* and *Arabidopsis.* Therefore, we hypothesized that *L. chinense* is evolutionarily closer to *O. sativa* than it is to Arabidopsis.

### Evolutionary rates of bzips in *L. chinense*

One of the most prevalent analyses in bioinformatics is the Ka/Ks analysis, which is crucial for the study of molecular evolution. To further understand the evolutionary relationships and processes among the LchibZIP families, we created isogenic maps (**Table S2**). For *Arabidopsis*, *Oryza*, and *L. chinense* the Ka, Ks, and Ka/Ks values for bZIP gene pairs were estimated. The Ka/Ks ratio for each of these orthologous gene pairs was less than 0.4. Showing that they experienced a significant negative selection during their evolution.

### *cis*-acting element analysis of LchibZIP promoters

**Figure 7:**
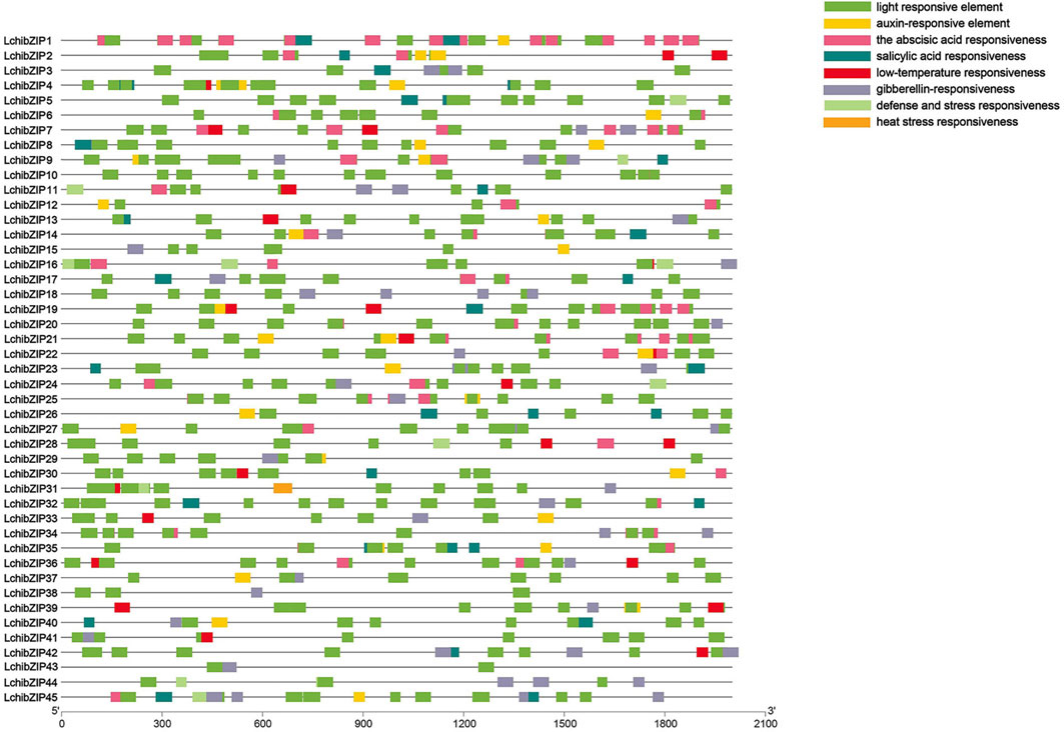
Cis-Acting element analysis of *L.chinense* bZIP gene promoters.

The *cis*-regulatory elements control dynamic networks of genes such as biotic and abiotic stress responses, hormone responses, and developmental processes. Therefore, to understand the transcriptional regulation and gene function of the bZIP genes, we searched the promoter regions of the 45 LchibZIPs (**Figure 7**, **Supplementary Figure S4**). The identified *cis*-elements were assigned into four different response groups: light, abiotic stress, plant hormone, plant growth and development. Our results showed that the abiotic stress responses had more *cis*-elements present in the promoter regions of all LchibZIPs, suggesting that they have an active role in the regulation of abiotic stress. Comparisons amongst the three abovementioned factors showed that light abiotic stress is expressed in all LchibZIPs. Specifically, the heat stress response element expressed only in LchibZIP31,and the low-temperature response elements were absent in some LchibZIP genes, for instance LchibZIP44 and LchibZIP 45 implying that certain bZIP genes in *L. chinense* regulate cold stress.

### Gene ontology (GO) annotation of LchibZIPs promoters

**Figure 8:**
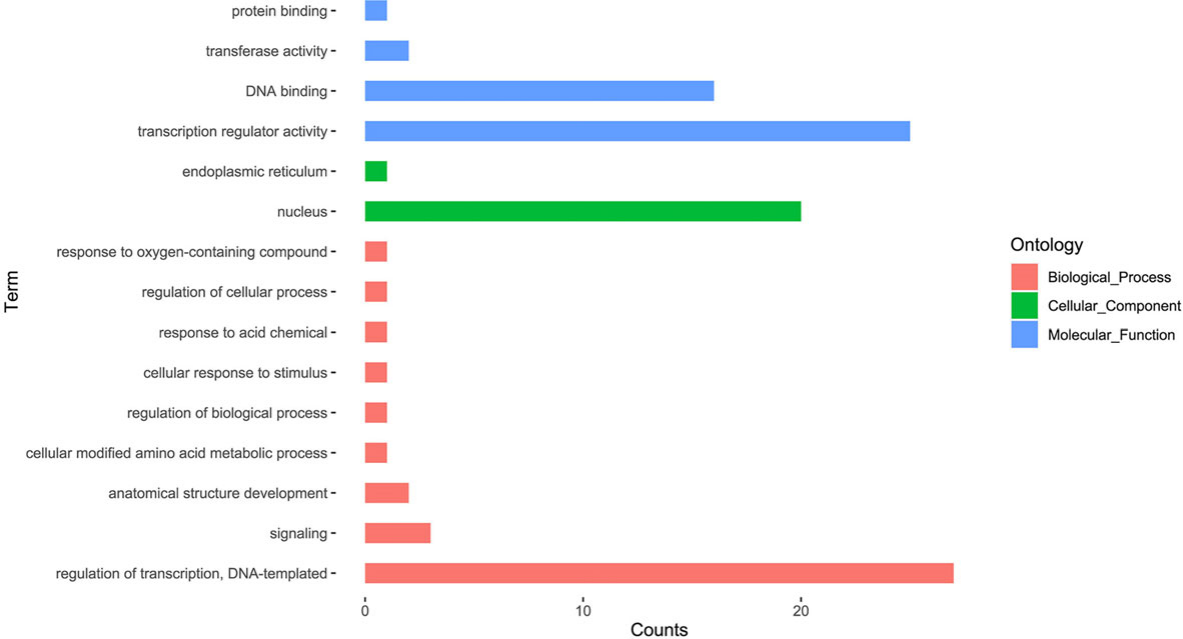
GO annotation results for the LchibZIPs from *L. chinense*.

Gene Ontology (GO) enrichment analysis provides an understanding of the possible functions of genes, we used the Blast2GO v5.2.5 to predict the biological functions of the identified LchibZIPs. The GO terms were categorized into three terms: the Cellular Component (CC), Biological process (BP), and Molecular Function (MF) (**Figure 8**). Nine BP terms were assigned to the *LcbZIPs*, these included: response to regulation of transcription (60%), signaling (6.6%), anatomical structure development (4.4%), cellular modified amino acid metabolic process (2.2%), regulation of biological process (2.2%), cellular response to stimulus (2.2%), response to acid chemical (2.2%), regulation of cellular process (2.2%), and response to oxygen-containing compound (2.2%). Four MFs were associated with LchibZIPs, these included: transcription regulator activity (55.5%), DNA binding (35.5%), transferase activity (4.4%), and protein binding (2.2%). Lastly, two CCs were assigned to the LchibZIPs: nucleus and endoplasmic reticulum, and 21 LchibZIPs were allocated for these two conditions. Multiple categories of LchibZIPs are shown in **Table S3**.

### bZIP protein interaction network in *L. chinense*

**Figure 9:**
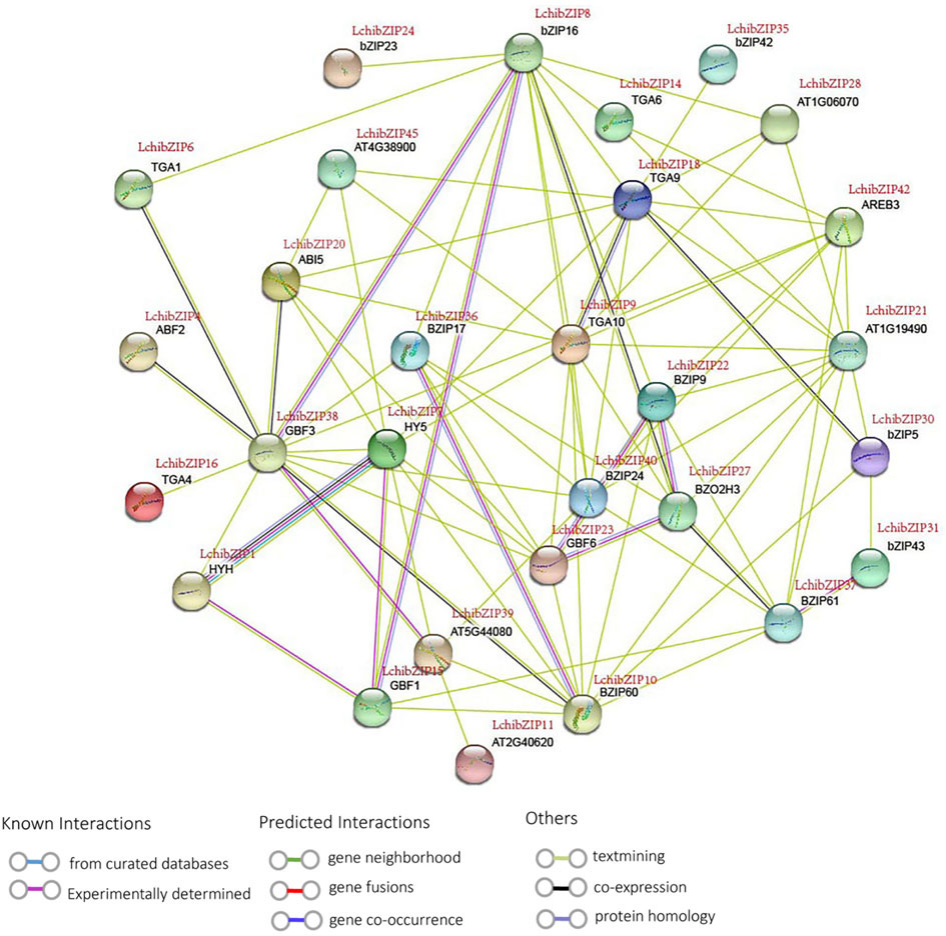
Protein interaction network of LchibZIPs from *L.chinense*. Purple lines indicate experimentally validated protein interactions; green lines indicate predicted protein interactions based on gene neighborhood; blue lines indicate predicted protein interactions based on gene co-occurrence; and black lines indicate protein coexpression.

To fully comprehend the possible interactions and functionality of the identified LchibZIP proteins, we mapped the LcbZIP protein interactions using STRINGv.10 database (https://string-db.org/) using the *A. thaliana* bZIPs as a homologous plant (**Figure 9**). 29 LchibZIP proteins interacted with each other, and their relation could be traced back to the phylogenetic relationships, suggesting that these proteins have similar functional roles. For instance, GBF3 (homologous to LchibZIP38), bZIP16 (LchibZIP8), and GBF1 (LchibZIP15) in group G; HY5 (LchibZIP7) and HYH (LchibZIP1) in group H had a strong interaction. Likewise, BZIP9 (LchibZIP22), BZO2H3 (LchibZIP27), GBF6 (LchibZIP23) and BZIP24 (LchibZIP40) had a strong interaction. These findings show that the interactions between LchibZIP proteins may be consistent with phylogenetic relationships.

Nonetheless, some proteins interacted to serve other functions; ABF2 (LchibZIP4), ABI5 (LchibZIP20), HY5 (LchibZIP7) and AREB3 (LchibZIP42) interacted and were involved in an abscisic acid-activated signaling pathway. TGA9 (LchibZIP18), TGA10 (LchibZIP9) were involved in the hydrogen peroxide mediated signaling pathway. bZIP17 (LchibZIP36) and bZIP24 (LchibZIP40) were involved in salt stress response. HYH (LchibZIP1) may play an vital role in controlling regulation of photomorphogenesis. With reference to the homologous *Arabidopsis* bZIP genes, we anticipated the functions of the LchibZIP proteins, which can gave us a deeper understanding of the recognition mechanisms between proteins, functional links, and the identification of new protein binding patterns.

### The differential expression analyses of LchibZIPs responding to cold stress

**Figure 10:**
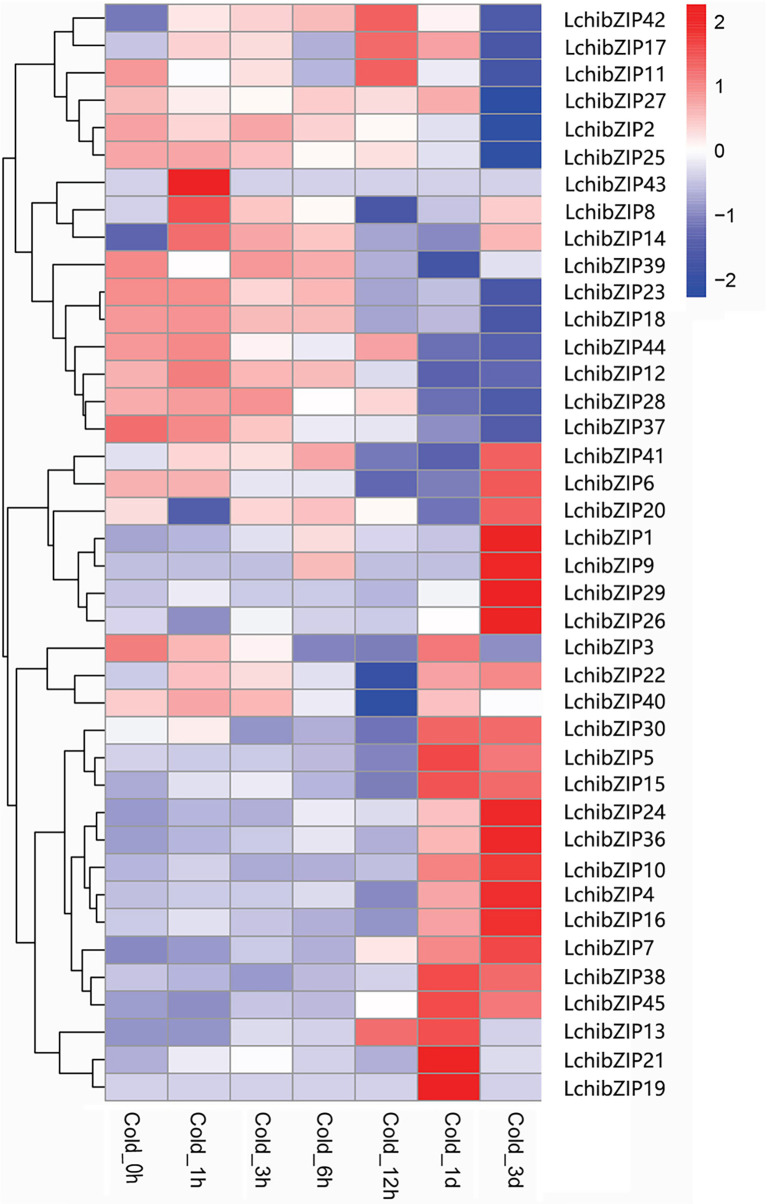
Expression profiles of LchibZIP genes. The RNA-sequence data was obtained from leaf of *L. chinense*.

To identify the bZIP genes involved in cold stress, we used the transcriptome data expression levels of LchibZIP genes at 1 h, 3 h, 6 h, 12 h, 1 d, and 3 d under low-temperature treatment (**Figure 10**). Four genes (LchibZIP4, LchibZIP7, LchibZIP24 and LchibZIP36) were markedly up-regulated under low-temperature stress, whereas the expression trends of seven genes, including LchibZIP2, LchibZIP12, LchibZIP18, LchibZIP23, LchibZIP25, LchibZIP27 and LchibZIP29, were significantly down-regulated. In addition, some genes were first up-regulated and then down-regulated after cold stress induction, such as LchibZIP5 and LchibZIP27. Several genes were first down-regulated and then up-regulated, such as LchibZIP10 and LchibZIP16, other genes were moderately expressed and had no significant changes. Generally, the results indicated a differential expression pattern of the LchibZIP genes, suggesting that LchibZIP genes regulate the cold stress at varying extends.

**Figure 11:**
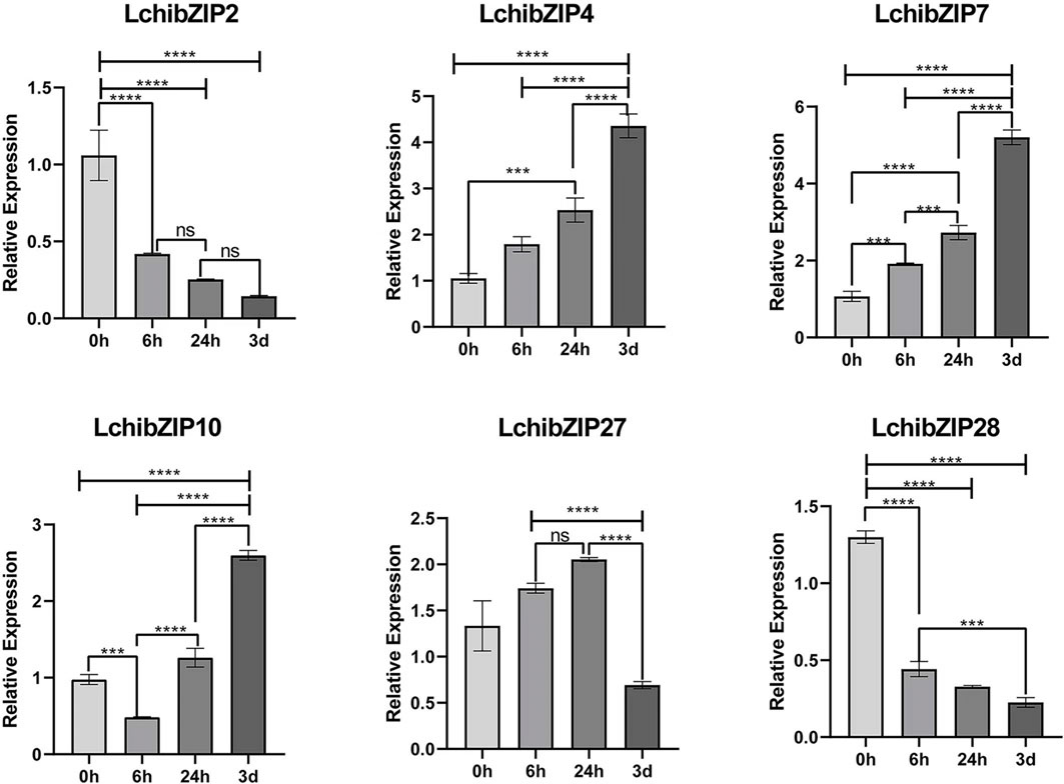
Expression analysis of 6 representative genes of *L.chinense* bZIP family under cold stress. The **** and *** indicate significant differences of p<0.0001 and p<0.001. The ns indicate no significant difference.

To validate the result of the transcriptome expression pattern, 15 LchibZIPs were selected for qRT-PCR assay (**Figure 11**). The result showed that six LchibZIPs were significantly expressed. That is, the expression patterns of LchibZIP4 and LchibZIP7 genes were elevated while, those of LchibZIP2 and LchibZIP28 were reduced. Additionally, the expression trend of LchibZIP10, was first decreased and then increased, and that of LchibZIP27 was characterized by an increase at first and then a decrease up to treatment termination. These findings concurred with the findings of the transcriptome gene expression profiling.

## Discussion

The *Liriodendron chinense* as Magnoliaceae plant species, play an important role both in the ecosystem balance and in the survival of the human society (Merkle, 1995). Like other plants, the *L. chinense* is affected by abiotic stresses, including low temperature stress. However, the bZIP genes are involved in the cold stress regulation in plants, and the bZIP transcription factors of magnolia have not yet been reported in the *L. chinense*. Although, they have been characterized in many plant species, and with varying gene family size (Wang et al., 2018; Li et al., 2020). The lower plants have the smallest gene family size (Liu and Chu, 2015). For instance; *Chlamydomonas reinhardtii* has a total of 17 bZIP genes while the earliest known moss on land, *Physcomitrella patens*, has 43 bZIP genes (Corrêa et al., 2008; Ji et al., 2018). In angiosperms, most plant genomes contain more than 40 *bZIP* genes (Baloglu et al., 2014; Liu and Chu, 2015). In this research we identified 45 LchibZIP genes, less than rose with 48 bZIP genes (Li et al., 2022b), *Juglans regia L.* has 88 *bZIP* genes (Zhang et al., 2022), and *Sedum plumbizincicola* has 92 *bZIP* genes (Lu et al., 2022).

The gene family size is to a greater extend controlled by genome and gene duplication events (Flagel and Wendel, 2009). Events such as tandem duplication and segmental duplication are essential for the expansion of gene family members during the evolution of plants (Ji et al., 2018). In this research, we showed the expansion of the LchibZIP genes was mainly due to the segmental duplication event. Deeper analysis showed that, 10 LchibZIPs were duplicated through segmental duplications, and only four gene pairs through tandem duplication, as demonstrated by synteny analysis of the LchibZIPs. Similarly, in *Juglans regia L.* bZIP genome, one pair of tandem repeats and 46 pairs of fragment repeats were identified (Zhang et al., 2022). In the pear bZIP genome, 12 pairs of tandem repeats and 65 pairs of fragment repeats were identified (Ma et al., 2021). In the apple bZIP genome, one pair of tandem repeats and 24 pairs of fragment repeats were identified (Wang et al., 2021). These findings support the fact that fragment repetition of the bZIP gene family is more frequent, which may be caused by domestication, and selected through a purified selection.

The evolutionary studies provide a trace of the genes and also may portray their relations with other plant species. In this research an evolutionary analysis of 10 species (*L. chinense*, *Chlamydomonas reinhardtii*, *Physcomitrella patens*, *Arabidopsi*s, *Vitis vinifera*, *Selaginella moellendorffii*, *Populus trichocarpa*, *Picea abies*, *Oryza sativa*, and *Musa acuminata*), showed that the 45 *bZIP* genes from *L. chinense* can be categorized into the 13 cluster groups by the phylogenetic tree, consistent with previous publications, suggesting that the *L. chinense* has conserved functional roles similar to homologs in the same cluster group (Pan et al., 2019). For example, subgroup D had LchibZIP6, LchibZIP9, LchibZIP14, LchibZIP16, and LchibZIP41 with AtbZIP11 among other protein, suggesting that these LchibZIPs may exhibit important pathogen defense roles (Hou et al., 2022; Jakoby et al., 2002).

The evolutionary studies may also cluster proteins based on the gene structure and motif arrangement similarities (Zhao et al., 2016). In this research LchibZIP proteins were also clustered based on gene structure and motif arrangements. Exons in LchibZIPs ranged in number from 1 to 10, and each subfamily had a comparable arrangement of exons and introns. According to our analysis, 11% of the genes lacked introns, which was comparable to the situations in soybean, rice, and sorghum (Banerjee and Roychoudhury, 2017). Nonetheless, a few genes lacked introns, while others lacked UTRs, demonstrating the variability of the gene structure important in functional analyses of significant *bZIP* candidate genes. Interestingly, our findings were inconsistent with the analysis of *Passiflora edulis* NAC protein interactions. This we related to the differences in the development processes of LchibZIP proteins and PeNAC proteins long-term domestication (Yang et al., 2022).

Current studies have all concurred in the involvement of bZIP *g*enes in the regulation of cold stress in plants. In *G. barbadense*, GbbZIP08, which may be involved in flavonoid production, have so far been discovered to have the highest expression rates in mature leaves under cold stress (Han et al., 2021). As a depressor of ABA signaling and a negative regulator of cold stress, the overexpression of the tea plant gene CsbZIP18 in *Arabidopsis* reduced cold tolerance (Yao et al., 2020). In the entire maize bZIP genome, ZmbZIP4 was discovered to be a positive regulator of plant abiotic stress response and engaged in maize root development (Ma et al., 2018). In kiwifruit, AchnABF1 plays an important role during cold stress (Jin et al., 2021). In this research, we found that 28 LchibZIPs had low temperature related cis-elements in the promoter region. In addition, we identified LchibZIP genes with low temperature responses at the transcriptome level, and further qRT-PCR validation of 6 LchibZIP genes also supported the involvement of LchibZIPs in cold regulation. Supporting previous publications that bZIPs mediate cold stress by interacting with various TFs.

In summary, a comprehensive genome-wide characterization of *L. chinense* bZIP family genes was conducted, and their expression levels under low-temperature stress were analyzed, revealing the importance of the bZIP genes in low-temperature response. Their interaction with one another, control signaling pathways, and activate defensive mechanisms in response to cold stress. Nonetheless, these findings are not exhaustive but rather provide a basis for further research on the function of LchibZIP genes, theoretical resources for future gene cloning and reference information for the regulation, structure, and function of the LchibZIP gene family.

## Conclusion

In the current study, we detected 45 bZIP gene sequences and analyzed genome data sets, taking into account phylogeny, gene architectures, and conserved motifs. Next, we used synteny analysis to determine where the genes were located on the chromosomes. The LchibZIP gene family was separated into 13 subsets by the phylogenetic tree, and the bZIP protein shared 20 common motifs with each subset. Gene structure and conservative motifs produced results that agreed with phylogenetic analyses. Intron counts varied greatly from 0 to 10, and members of the same group also shared similarities in gene structure and the number of exons and introns. 45 LchibZIPs were found to be distributed across 16 chromosomes using homology analysis. Additionally, 4 pairs of tandem repeats and 10 pairs of fragment repeats were found, and they all had Ka/Ks ratios of less than 0.4, suggesting that they may have undergone a strong purification selection during evolution. We generated significant research suggestions for further investigation of the new roles of LchibZIPs from protein-protein interactions and gene ontology annotations of the LchibZIPs. The six genes were then shown by qRT-PCR to be connected to cold stress, and the overexpression of LchibZIP7 and LchibZIP4 improved the plant’s ability to withstand cold temperatures. Thorough research revealed that the bZIP gene family, which includes homologous bZIP members between *Arabidopsis* and *L. chinense*, may play a number of roles in a variety of biological processes. In controlling plant development and stress response, *L. chinense* might perform a conservative role. Further research on the biological role of genes can be supported by these findings.

## Data availability statement

The datasets presented in this study can be found in online repositories. The names of the repository/repositories and accession number(s) can be found in the article/**Supplementary Material**.

## Author contributions

JW and LY designed the experiments. ML, DH, YL, TM, BA, and WZ accomplished the experiments and analyzed the data. ML and DH wrote the manuscript. All authors reviewed and approved the manuscript.

## Funding

This research work was funded by the National Natural Science Foundation of China (No. 31971682), the Research Start-up Fund for High-Level and High-Educated Talents of Nanjing Forestry University, and the Jiangsu Agricultural Science and Technology Innovation Fund (SCX (22)31207).

## Acknowledgments

We appreciate the time and effort put in by the reviewers, editors, and funders of this study in providing helpful advice that enabled us do work efficiently.

## Conflict of interest

The authors declare that the research was conducted in the absence of any commercial or financial relationships that could be construed as a potential conflict of interest.

## Publisher’s note

All claims expressed in this article are solely those of the authors and do not necessarily represent those of their affiliated organizations, or those of the publisher, the editors and the reviewers. Any product that may be evaluated in this article, or claim that may be made by its manufacturer, is not guaranteed or endorsed by the publisher.
