# Supplementary material for: The bZIP transcription factors in Liriodendron chinense: Genome-wide recognition, characteristics and cold stress response
Source: Front Plant Sci. 2022 Nov 7;13:1035627. doi: 10.3389/fpls.2022.1035627 (PMC9676487; doi:10.3389/fpls.2022.1035627)
Supplement: Supplementary file 4 [file DataSheet_2.pdf]

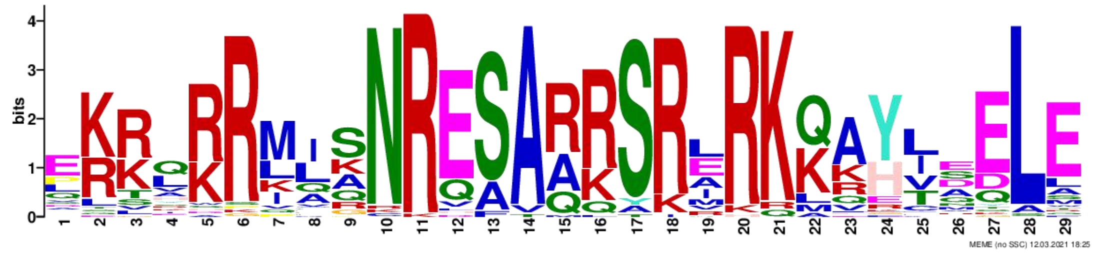

Logo1

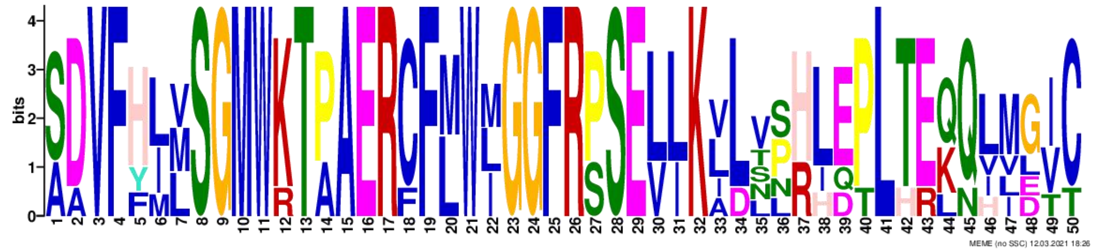

Logo2

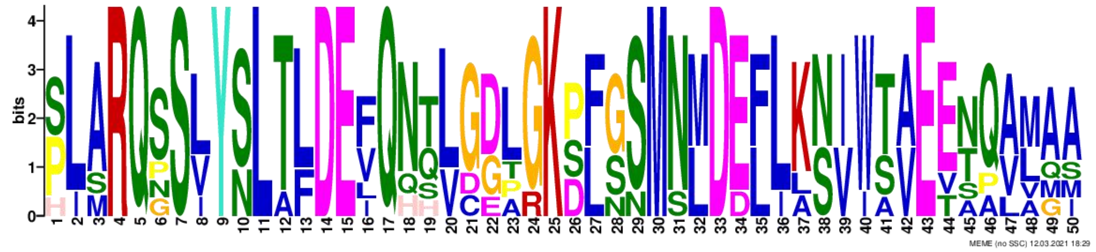

Logo3

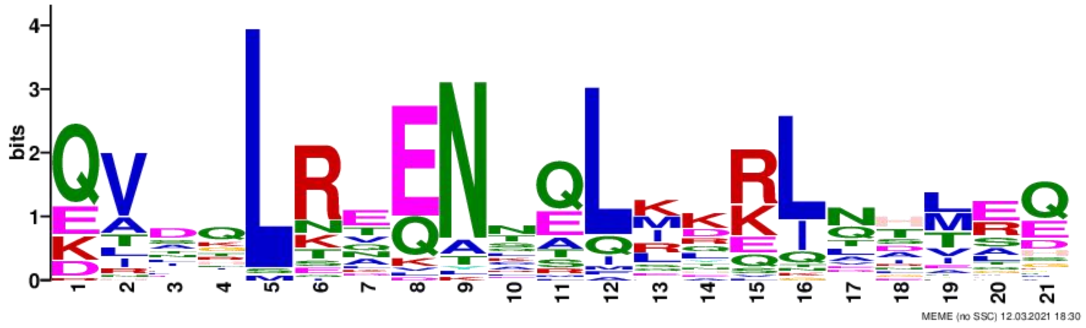

Logo4

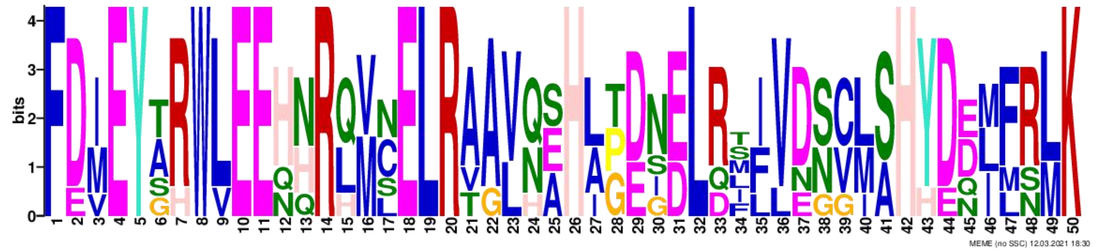

Logo5

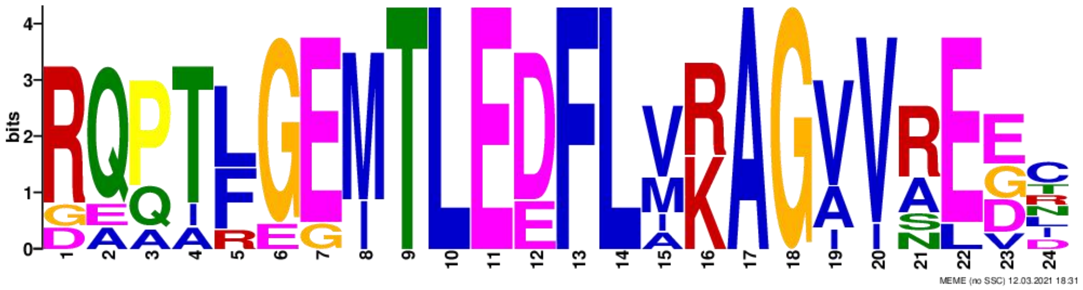

Logo6

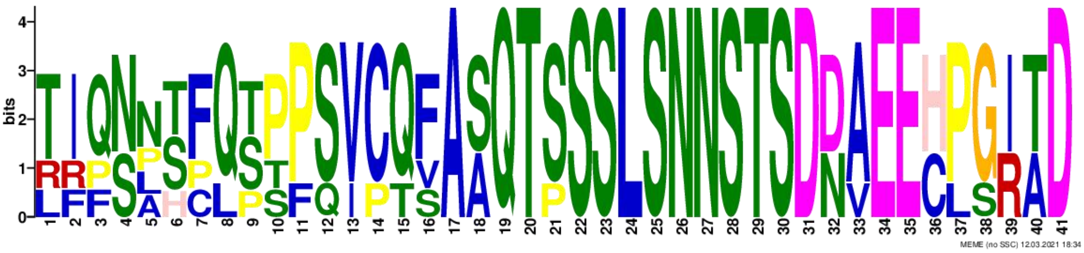

Logo7

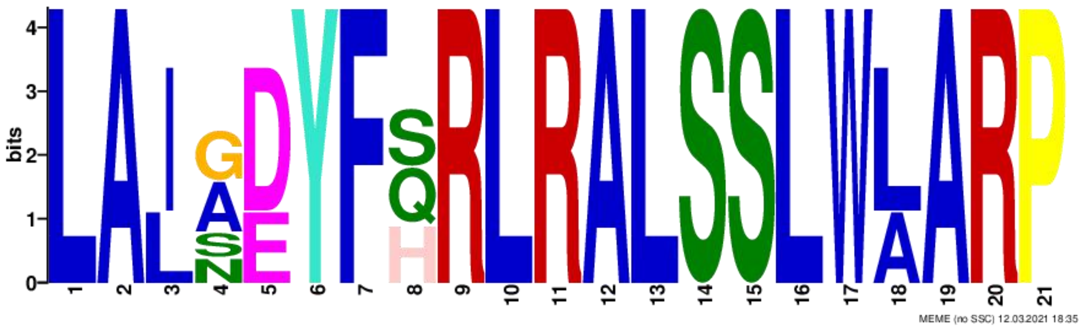

Logo8

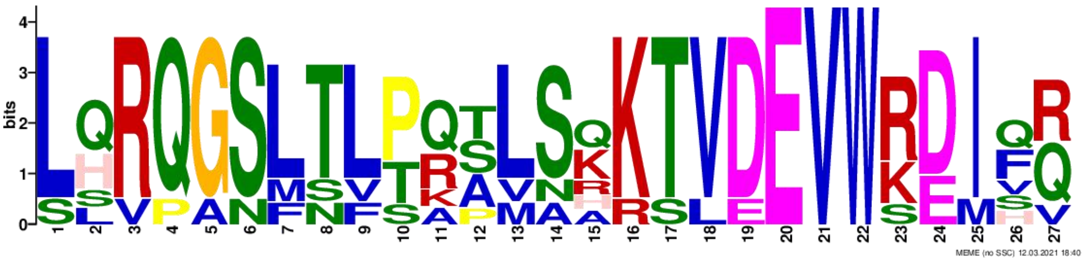

Logo9

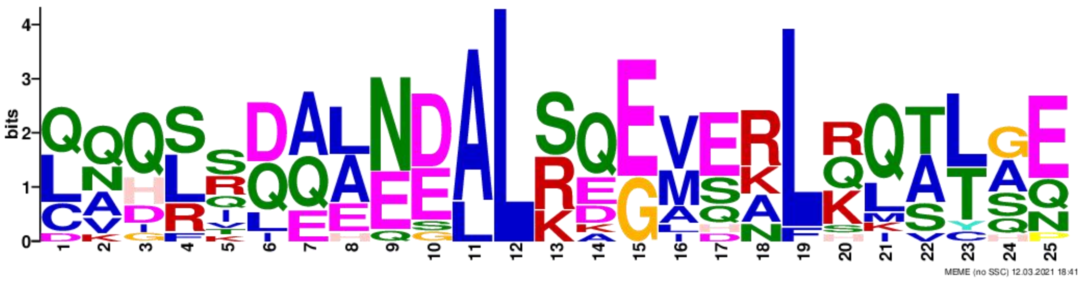

Logo10

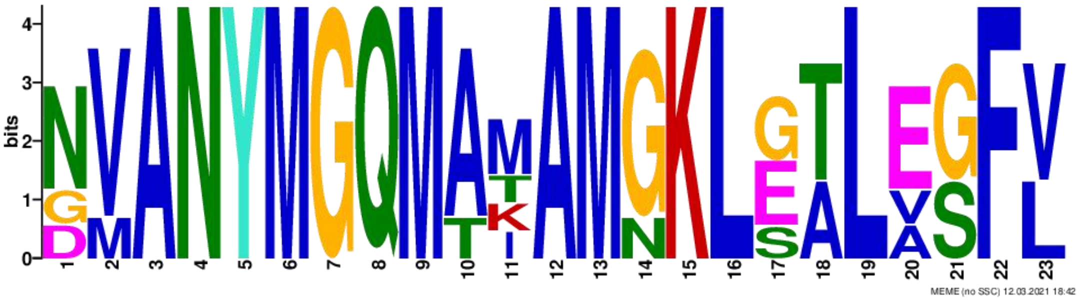

Logo11

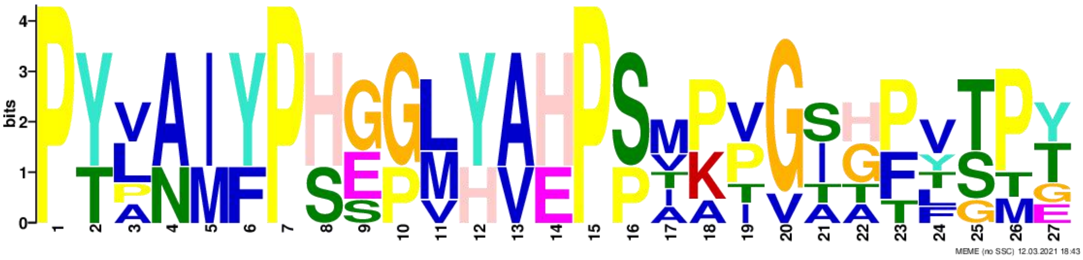

Logo12

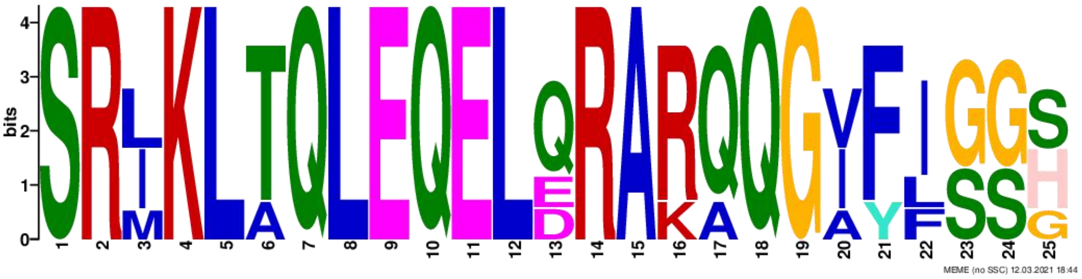

Logo13

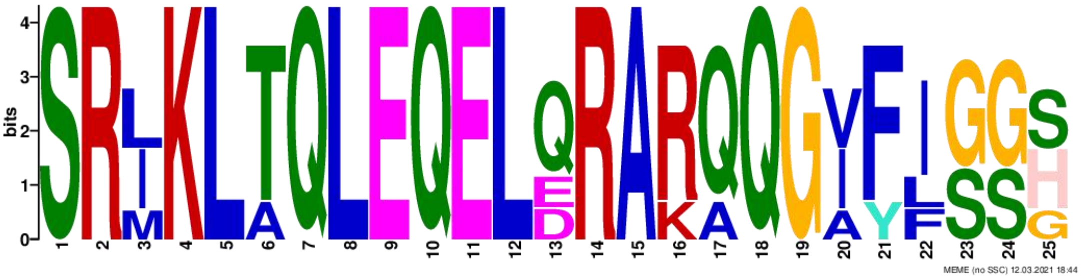

Logo14

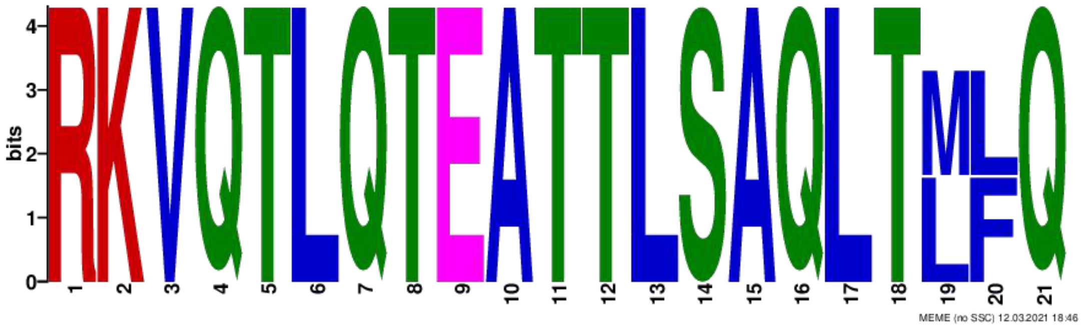

Logo15

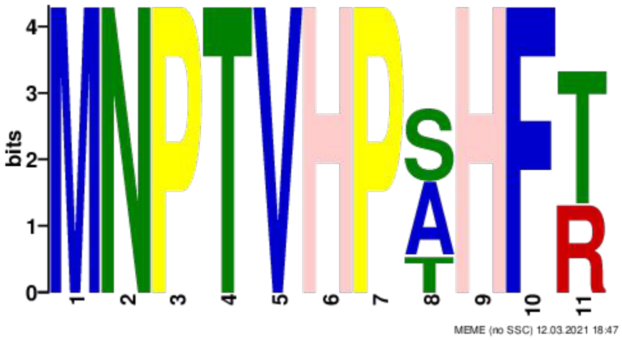

Logo16

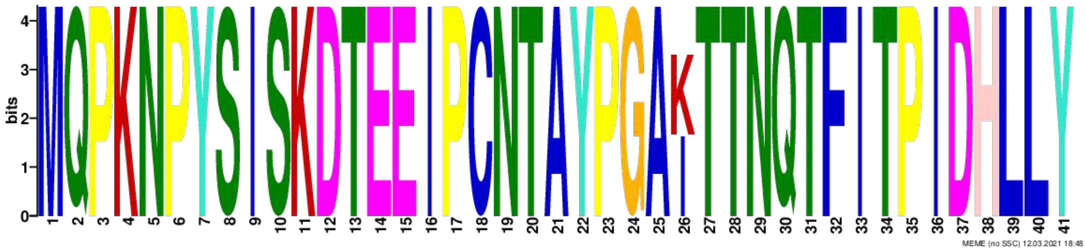

Logo17

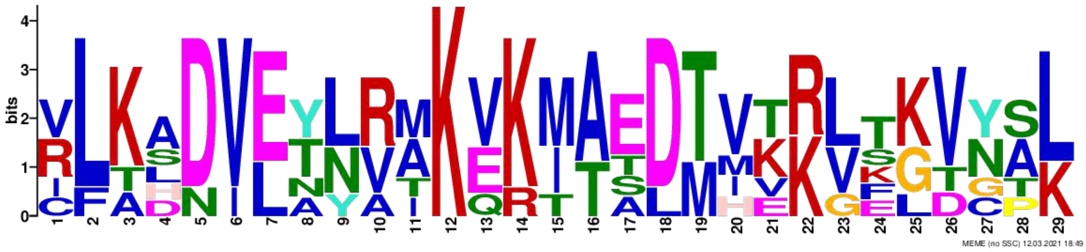

Logo18

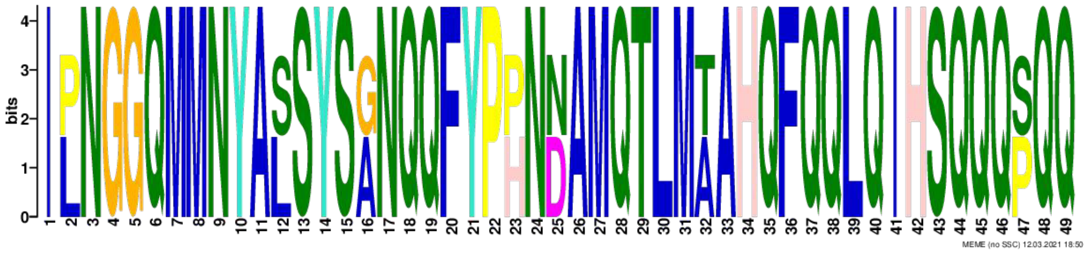

Logo19

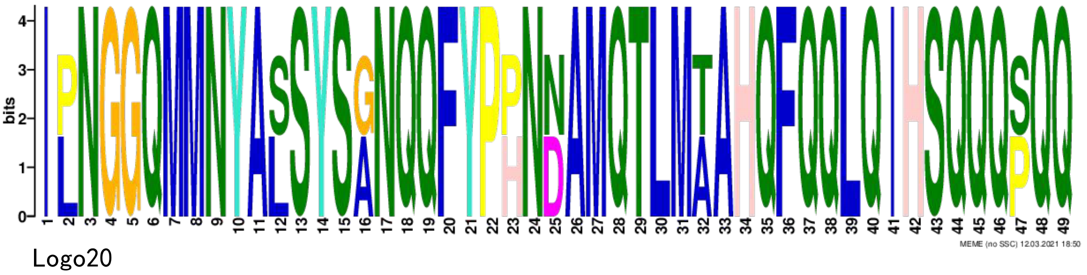

Logo20

Figure s1.Seqlogo of conserved motifs in LchibZIPs.
