# Supplementary material for: The bZIP transcription factors in Liriodendron chinense: Genome-wide recognition, characteristics and cold stress response
Source: Front Plant Sci. 2022 Nov 7;13:1035627. doi: 10.3389/fpls.2022.1035627 (PMC9676487; doi:10.3389/fpls.2022.1035627)

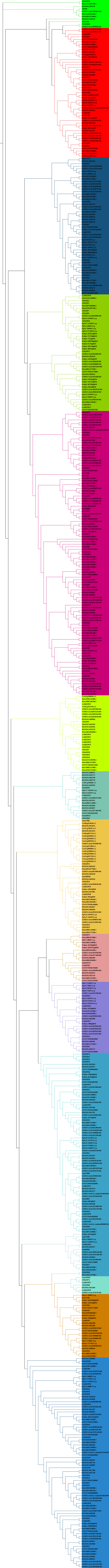

Supplement: Supplementary file 3 [file DataSheet_1.pdf]
